# Central as well as Peripheral Attentional Bottlenecks in Dual-Task Performance Activate Lateral Prefrontal Cortices

**DOI:** 10.3389/fnhum.2016.00119

**Published:** 2016-03-16

**Authors:** André J. Szameitat, Azonya Vanloo, Hermann J. Müller

**Affiliations:** ^1^Division of Psychology and CUBIC, Department of Life Sciences, Brunel UniversityLondon, UK; ^2^Department of Psychology, Ludwig Maximilians UniversityMunich, Germany

**Keywords:** lateral prefrontal cortex, executive functions, psychological refractory period (PRP), response selection bottleneck, response initiation bottleneck, multitasking, functional magnetic brain imaging (fMRI)

## Abstract

Human information processing suffers from severe limitations in parallel processing. In particular, when required to respond to two stimuli in rapid succession, processing bottlenecks may appear at central and peripheral stages of task processing. Importantly, it has been suggested that executive functions are needed to resolve the interference arising at such bottlenecks. The aims of the present study were to test whether central attentional limitations (i.e., bottleneck at the decisional response selection stage) as well as peripheral limitations (i.e., bottleneck at response initiation) both demand executive functions located in the lateral prefrontal cortex. For this, we re-analyzed two previous studies, in which a total of 33 participants performed a dual-task according to the paradigm of the psychological refractory period (PRP) during functional magnetic resonance imaging (fMRI). In one study (*N* = 17), the PRP task consisted of two two-choice response tasks known to suffer from a central bottleneck (CB group). In the other study (*N* = 16), the PRP task consisted of two simple-response tasks known to suffer from a peripheral bottleneck (PB group). Both groups showed considerable dual-task costs in form of slowing of the second response in the dual-task (PRP effect). Imaging results are based on the subtraction of both single-tasks from the dual-task within each group. In the CB group, the bilateral middle frontal gyri and inferior frontal gyri were activated. Higher activation in these areas was associated with lower dual-task costs. In the PB group, the right middle frontal and inferior frontal gyrus (IFG) were activated. Here, higher activation was associated with higher dual-task costs. In conclusion we suggest that central and peripheral bottlenecks both demand executive functions located in lateral prefrontal cortices (LPFC). Differences between the CB and PB groups with respect to the exact prefrontal areas activated and the correlational patterns suggest that the executive functions resolving interference at least partially differ between the groups.

## Introduction

The human brain is generally considered to be capable of massive parallel processing. While this is certainly true in many respects, there are also severe limitations to this parallelism which only allow for strictly serial processing of information. One such fundamental limitation is the seemingly easy ability to select a response for a stimulus: The selection of a response to one stimulus (e.g., if the tone has a low pitch press the left middle finger, if it has a high pitch, press the left index finger) delays the selection of another response to another stimulus by several hundreds of milliseconds—a phenomenon referred to as “psychological refractory period” (PRP; Telford, 1931; Welford, 1952). In more detail, the central stage of the response selection, i.e., the decisional process linking stimuli to their appropriate responses, cannot work in parallel (Pashler, 1984). Therefore, it constitutes a central bottleneck (CB), i.e., a central attentional limitation (Pashler, 1994; Marois and Ivanoff, 2005; Tombu et al., 2011). Because this bottleneck is virtually invincible, it strongly affects human information processing and has widespread implications for everyday life (Pashler, 1993), such as people’s ability for multitasking in workplaces, operating machinery, or driving a car.

However, even more basic tasks which lack a response selection stage can be subject to processing limitations. For instance, in simple response tasks there is only a single response required to one potential stimulus (e.g., if there is a tone press the left index finger). In other words, there are no sets of stimuli and responses to select from, and consequently there is no response selection stage. Nevertheless, even such a simple response task delays another simple response task again by several hundreds of milliseconds (Karlin and Kestenbaum, 1968; De Jong, 1993; Schubert, 1999). In this example, it has been argued that the bottleneck is not central (response selection), but peripheral at the stage of response initiation (De Jong, 1993; Schubert, 1999). Due to its very basic nature, it has even more profound implications for everyday life.

One crucial aspect concerning the existence of such bottlenecks in the information processing chain is that they result in the need for executive functions that resolve the ensuing interference between the two tasks (Frith and Done, 1986; Szameitat et al., 2002; Marois and Ivanoff, 2005; Schubert, 2008). Executive functions are assumed to minimize interference by scheduling the order in which the tasks are processed, interrupting the task which has to wait, e.g., by inhibiting it, switching to the interrupted task, and reinstating the interrupted task once bottleneck processing for the first task has finished (De Jong, 1995; Meyer and Kieras, 1997; Logan and Gordon, 2001)^1^. Notably, these executive functions are required only when two tasks have to be performed concurrently as a dual task, but not when they are performed individually as single tasks. Consequently, multitasking not only results in performance decrements but also demands executive functions.

These executive functions, such as switching and inhibition, require mental operations. For instance, switching between task sets involves the retrieval of stimulus-response mappings from long-term memory and their loading into working memory (Monsell, 2003), while inhibition involves the active suppression of other representations (such as memory contents or response intentions; Konishi et al., 1999; Bunge et al., 2001; Aron et al., 2003). These mental operations are likely to increase neural processing and therefore also the blood-oxygen-level-dependent (BOLD) response. Consequently, we predicted higher BOLD responses in the dual-task as compared to the single-tasks.

The prediction of additional activation in the dual-task depends on the presence of a bottleneck. In the current manuscript, we take the observation of a PRP effect, i.e., the prolongation of the second task’s response times in the dual-task as compared to the single-task, as evidence that a bottleneck has been present (Pashler, 1984, 1994). Thus, observation of prolonged response times in the second task can be seen as a prerequisite for the hypothesis that additional executive functions are needed in the dual-task, which in turn should result in additional functional magnetic resonance imaging (fMRI) activation.

For CBs at the response selection stage, the executive functions resolving dual-task interference have been localized mainly in the left and right lateral prefrontal cortices (LPFC), with a tendency for stronger involvement of the left LPFC (Szameitat et al., 2002; Schubert and Szameitat, 2003; Stelzel et al., 2009). However, for peripheral bottlenecks (PBs) at the response initiation stage, the executive functions resolving dual-task interference have not been localized as yet (but see Herath et al., 2001). Thus, it is unclear whether PBs demand the LPFC and if they do, whether they involve the LPFC in a comparable fashion to CBs^2^.

The current study aimed at resolving these open questions. For this, we re-analyzed the results of two previous studies of ours (no results of either study have been published thus far). In one study, participants performed a PRP dual task based on two-choice response tasks (e.g., deciding by button press whether a visually presented face was male or female), which are known to suffer from a central bottleneck (CB group). In the other study, participants performed a PRP dual task based on simple-response tasks (e.g., press a button as soon as a face is presented), which are known to suffer from a peripheral bottleneck (PB group). Our first aim was to test the hypothesis that a PB in a PRP paradigm also results in dual-task specific activation in the LPFC which may be linked to executive functions. As outlined above, such executive functions should be present only in dual-task, but not in single-task performance. Consequently, we define areas as being specific to bottleneck processing when they exhibit over-additivity as compared to the summed single-tasks (contrast: PRP Dual Task – Single Task 1 – Single Task 2; [1 −1 −1]; for an in-depth analysis of potential contrasts in dual-task studies, see Szameitat et al., 2011).

Our second aim was to compare the areas identified for PBs with those of CBs. However, note that a direct comparison of the CB and PB group seems inappropriate, because the data were derived from two different fMRI scanners which may differ in their physical properties, such as signal-to-noise ratios. Consequently, differences in a direct statistical comparison of the CB and PB groups might be caused either by scanner characteristics or by the experimental manipulation (see “Discussion” Section for a further discussion). Instead, we compared the results qualitatively by visual inspection of the activated areas. Therefore, this research question should be considered as a pilot study of exploratory character.

Regarding this exploratory comparison of the CB and PB groups, it seems plausible to assume that central and peripheral bottlenecks result in largely comparable demands to coordinate task processing. For instance, for CBs, it has been shown that controlling the order in which the tasks are processed by the bottleneck is one central coordination requirement localized in the LPFC (Szameitat et al., 2006; Stelzel et al., 2008). Note that in PRP paradigms (like in the present study), participants usually have to respond to the tasks in a certain order. Such order control may involve pre-setting the bottleneck to the task which has to be processed first and monitoring whether the tasks are indeed processed in the correct order (De Jong, 1995; Luria and Meiran, 2003). In simple-response PRP tasks, these demands are present in the same way, so that we expected at least partially overlapping areas in the LPFC to be associated with the coordination of task processing at central and peripheral bottlenecks. Other demands, however, may be specific to either central or peripheral bottlenecks. For instance, demands to load stimulus-response mappings into working memory are likely to be higher when two-choice-response tasks are used (CB group) as compared to simple-response tasks (PB group; Stelzel et al., 2008). Consequently, we also expected differences between the CB and PB groups with respect to the exact neural correlates of bottleneck processing.

A third aim was to characterize the involved executive functions in more detail. For this, we first determined the behavioral costs of the bottleneck by comparing the mean response times of the second task in the PRP-dual task with the (averaged) response times of the single tasks. This measure of behavioral costs reflects not only the PRP, i.e., the waiting of the second task for the first task to being processed, but also the executive functions which schedule the task processing at the bottleneck (De Jong, 1995; Luria and Meiran, 2003; Marois and Ivanoff, 2005; Sigman and Dehaene, 2005). In a second step, we correlated these behavioral costs with the dual-task specific LPFC activation. Crucially, LPFC activation can be linked to behavioral costs in at least two different ways, depending on the exact nature of the interference in the dual-task and the particular executive functions involved in its resolution. For instance, De Jong (1993) and Logan and Gordon (2001) suggested that one type of interference in PRP dual-tasks is crosstalk between the two tasks and that this crosstalk can be resolved by inhibition of one of the two tasks. The amount of crosstalk varies between participants (Herath et al., 2001) and it is typically assumed that: (a) higher crosstalk demands more executive functions to resolve it and (b) consequently results in higher behavioral costs (Logan and Gordon, 2001). Thus, if crosstalk is the major source of interference in a PRP dual-task, then increased behavioral costs should be associated with higher LPFC activation (i.e., a positive correlation). Data of Frith and Done (1986) and Herath et al. (2001) suggest that crosstalk is indeed the main source of interference specifically in simple-response tasks. Consequently, we predicted a positive correlation between costs and LPFC activation for the PB group.

A second way in which behavioral costs and LPFC activation can be linked is in form of a negative correlation, i.e., higher activation is associated with lower costs. Such a pattern can be predicted when task preparation is taken into account, because task preparation incorporates mental processes which take place before the actual task performance. For PRP dual-tasks it has been shown that participants automatically prepare task processing by switching the bottleneck to the first expected task, that this takes place even before the first stimulus is shown, and that better preparation reduces dual-task costs (De Jong, 1995; Luria and Meiran, 2003). More and better preparation involves more mental demands and therefore increases LPFC activation (Herath et al., 2001; Brass and von Cramon, 2002). It has been argued that such preparatory processes are more relevant in choice tasks, potentially because it is more demanding to load and prepare several specific stimulus-response mappings (e.g., male face = left finger; female face = right finger) as a compared to the basic and simplistic rules required in simple-response tasks (e.g., any visual stimulus = finger response; Frith and Done, 1986; Schubert, 1999; Monsell, 2003). Consequently, we predicted a negative correlation between costs and LPFC activation for the CB group.

To summarize, for the PB group we predicted that increased dual-task costs are associated with increased LPFC activation because higher interference in form of crosstalk results in increasing demands on coordinative executive functions in the LPFC (Herath et al., 2001). For the CB group we predicted that increased dual-task costs are associated with decreased LPFC activation because the increased costs indicate poorer advance preparation by the participants with consequently lower involvement of executive functions. We aim to correlate the individual beta-values at LPFC peak voxels in the CB group (central bottleneck; choice response task) and the PB group (peripheral bottleneck; simple response tasks) with the respective individual dual-task costs. A different correlational pattern would indicate that central and peripheral bottlenecks demand executive functions differently, even if they activate comparable areas of the LPFC.

## Materials and Methods

### Participants

For the present report, we combined the datasets of two studies conducted in different contexts. In the PB group, 16 (7 female) participants (aged 19–38; mean 24 years) took part after giving written informed consent in accordance with the Declaration of Helsinki. This study was part of the thesis for a MSc in Functional Neuroimaging^3^ by one of the co-authors (AV) and was approved by the Brunel University (London, UK) ethics committee. In the CB group, 17 (9 female) participants (aged 22–28; mean 24 years) took part after giving written informed consent in accordance with the Declaration of Helsinki. This study was conducted as a practical teaching exercise in the MSc in Neurocognitive Psychology program^4^ and was approved by the Ludwig Maximilians University (Munich, Germany) ethics committee.

### Task and Procedure

Participants were lying in the fMRI scanner in supine position, holding two MRI compatible response pads in their hands. Task instructions and stimuli were presented on a projection screen via a mirror system. There were four conditions relevant to the present study, presented in an fMRI blocked design. Since this report is based on two different studies, there were slight differences in the implementation of the tasks. We first describe the conditions as presented to the CB group (i.e., PRP task based on choice-response tasks).

In the CB group, a trial in the condition auditory single-task (AUD-ST) started with the auditory presentation of syllables for 345 ms (on average) via MRI-suitable headphones. Participants had to press a button with their left-hand middle finger if the syllables were /yaya/, and a button with their left-hand index finger if the syllables were /haha/. From the start of stimulus presentation, participants had 3040 ms to respond. After this time, either error feedback was presented (“error”) or a fixation cross was presented for 180 ms. Thus, one trial lasted 3220 ms in total. The visual single task (VIS-ST) was identical except that participants were presented with either a male or a female face in the center of the screen for 345 ms. Participants were instructed to press the right-hand index finger when the face was male, and the right-hand middle finger if it was female. There were two dual-task conditions (DT) in which both stimuli were presented at the same time (stimulus-onset-asynchrony, SOA, of 0 ms). In one condition, participants were instructed to respond to the auditory task first (DT-AV); in the other condition, they were instructed to respond to the visual task first (DT-VA). If participants responded in the wrong order, they received an error-feedback message (“error”). In all other respects, this condition was identical to the single tasks. Finally, there was a resting-baseline (Rest) condition lasting 29 s during which participants were instructed to fixate a cross on the screen.

In the CB group, tasks were presented in blocks of nine trials, lasting 29 s (fMRI block design). Blocks were separated by a 4 s interval during which the instructions for the upcoming block were presented (e.g., “Dual-task, Syllable => Face”). Each condition was presented six times in an individually pseudo-randomized order. In this study, there were five further conditions not relevant to the current report.

Next, we describe the conditions as presented to the PB group (i.e., a PRP task based on simple-response tasks). Visual (faces) and auditory (syllables) stimuli were identical to the CB group. Participants were instructed to press a button with their left-hand index finger if they heard a syllable (irrespective of whether it was /yaya/ or /haha/), and to press a button with their right-hand index finger if they saw a face (irrespective of whether it was male or female). There were no further auditory stimuli. Except for the fixation cross and the error feedback message, there were also no further visual stimuli. In the PB group, a trial in the AUD-ST condition started with a fixation cross for 345 ms, after which the syllables were presented for 345 ms. From the start of the first stimulus, participants had 1855 ms to respond. After the response, either an error-feedback message (“error”) or a fixation cross was presented for 300 ms. Thus, a trial lasted 2500 ms in total. There was only one dual-task condition (DT-AV) in which participants had to respond to the auditory task first. The SOA varied randomly between 0 ms and 400 ms (mean 200 ms; SD 115 ms) to avoid perfect predictability of the onset of the second stimulus and anticipatory responses by the participants (Frith and Done, 1986; Schubert, 1999). Finally, there was a Rest condition lasting 25 s.

In the PB group, tasks were presented in blocks of 10 trials, lasting 25 s (fMRI block design). Blocks were separated by a 5 s interval during which the instructions were presented. Each condition was presented 8 times in a pseudo-randomized order. In this study, there were two further conditions not relevant to the current report.

### MRI Procedure

Imaging of the CB group was carried out at the Max Planck Institute of Psychiatry, Munich, Germany, using a 3T GE MR-750 scanner equipped with a 12-channel headcoil. Participants were supine on the scanner bed and cushions were used to reduce head motion. Thirty-two axial slices (192 mm × 192 mm field of view (FOV), 64 × 64 matrix, 3 mm slice thickness, no gap, interleaved slice acquisition, voxel size 3 mm × 3 mm × 3 mm) were acquired using a BOLD-sensitive gradient echo EPI sequence (TR 2s, TE 30 ms, 90° flip angle). High-resolution whole-brain anatomical images were acquired from each participant using a T1-weighted FSPGR (fast spoiled grass) sequence (TR 7.1 s, 128 slices, TE 2.2 ms, 12° flip angle, 240 mm × 240 mm FOV). Two functional runs with 516 volumes each were acquired, with each volume sampling all 32 slices.

Imaging of the PB group was carried out at CUBIC (Royal Holloway University London, UK)^5^, using a 3T scanner (Trio, Siemens, Erlangen, Germany) equipped with a 12-channel array head coil. Participants were supine on the scanner bed, and cushions were used to reduce head motion. Thirty-five axial slices (192 mm × 192 mm FOV, 64 × 64 matrix, 3 mm × 3 mm in-plane resolution, 3 mm thickness, no gap, interleaved slice acquisition) were acquired using a BOLD-sensitive gradient echo EPI sequence (TR 2.5 s, TE 31 ms, 85° flip angle). High-resolution whole-brain images were acquired from each participant using a T1-weighted MPRAGE sequence (TR 1830 ms, TE 3.03 ms, 11° flip angle, 160 slices, 256 mm × 256 mm FOV, 1 mm × 1 mm × 1 mm voxel size). One functional run with 577 volumes was acquired.

### Data Analysis

Data were analyzed using SPM12^6^. First, the origin of the anatomical and functional images was manually set to the anterior commissure and all images were reoriented. To correct for movements, all functional volumes were spatially realigned to the first functional volume, and signal changes due to head motion and magnetic field inhomogeneities were corrected (Realign and Unwarp; Andersson et al., 2001). Anatomical and functional images were normalized to MNI space using unified segmentation. Finally, the functional data were spatially smoothed using a Gaussian kernel with a FWHM of 8 mm. Normalization and registration success was validated by visual inspection.

Both studies employed block-designs, and statistical analysis was based on a voxel-wise least-squares estimation using the general linear model for serially autocorrelated observations (Friston et al., 1994). Temporal high-pass filters with cut-off frequencies of 1/165 Hz (CB group) and 1/180 Hz (PB group) were applied. Individual contrast maps were calculated for all contrasts of interest (see “Results” Section), and the second-level analysis was based on random-effects one-sample *t*-tests. Because the data of the CB and PB groups were derived from two different scanning sites, we did not compare them directly, e.g., by using two-sample *t*-tests. All resulting *t*-maps were thresholded at *p* < 0.05 (FWE corrected for multiple comparisons). For display purposes, graphs were thresholded at *p* < 0.00005 (uncorrected). All stereotaxic coordinates are reported in MNI space. Anatomical locations and Brodmann’s areas (BAs) were preferentially determined using the SPM Anatomy toolbox (Eickhoff et al., 2005) or alternatively the Automated Anatomical Labeling toolbox (Tzourio-Mazoyer et al., 2002).

## Results

### Behavioral Results

**Figure 1:**
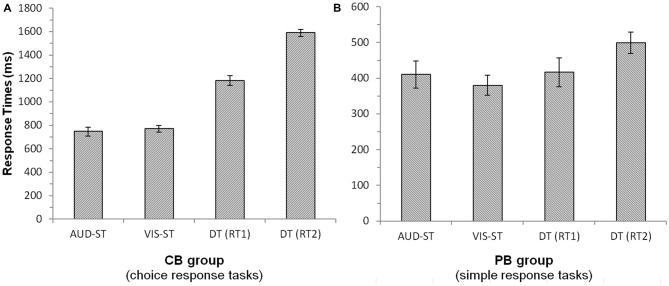
**Response times of the central bottleneck (CB, A) group and the peripheral bottleneck (PB, B) group.** Error bars denote 95%-confidence intervals (Loftus and Masson, 1994). Note the different scales of the two panels. Notes: AUD-ST/VIS-ST, auditory/visual single-task; DT, dual-task; RT1/RT2, response time of the first/second task in the dual-task.

Averaged response times were calculated based on correct-response trials only. Unless otherwise noted, paired-samples *t*-tests were conducted. For the CB group (Figure 1A), response times of the first task (RT1) in the dual task were significantly longer as compared to the single-task response times (average of the two DT (1182 ms) vs. average of the two single-tasks (761 ms), *t*_(16)_ = 14.58, *p* < 0.001). The presence of a bottleneck delays in particular the second task. This is supported by the finding that in the DT, response times of the second task (RT2; 1590 ms) were significantly longer than response times of the first task (1182 ms; *t*_(16)_ = 14.36, *p* < 0.001). Auditory (747 ms) and visual (769 ms) single tasks did not differ significantly from each other (*t*_(16)_ = 0.92, *p* = 0.374). The error rates showed a similar pattern, with participants making significantly more errors in the dual task (10.6%) than in the single tasks (3.9%; *t*_(16)_ = 4.01, *p* < 0.01).

For the PB group (Figure 1B), RT1s in the dual task (417 ms; always auditory task) did not differ significantly from the auditory single-task RTs (410 ms; *t*_(15)_ = 0.772, *p* = 0.452). However, RT2s in the dual task (499 ms; always visual task) were significantly longer than RT1s in the dual task (417 ms; *t*_(15)_ = 5.3, *p* < 0.001) as well as the visual single task RTs (380 ms; *t*_(15)_ = 5.06, *p* < 0.001). This nicely illustrates the deferment of RT2 as a consequence of a PB. Auditory (410 ms) and visual (380 ms) single-task RTs did not differ significantly from each other (*t*_(15)_ = 1.64, *p* = 0.122). Error rates showed more errors in the dual-task condition (5.5%) as compared to the single-task conditions (1.4%; *t*_(15)_ = 3.05, *p* < 0.01).

Taken together, we found profound and significant deferments of responses to the second task in the CB as well as the PB group, which is strong evidence for the presence of processing bottlenecks.

### fMRI Results

In both groups, we tested for activation specific to the dual task (i.e., presence of a bottleneck) by subtracting the sum of the single tasks from the dual task by using the contrast (DT – AUD-ST – VIS-ST, i.e., [1 −1 −1]; Szameitat et al., 2011). We focus in particular on lateral prefrontal areas.

#### CB Group

**Figure 2:**
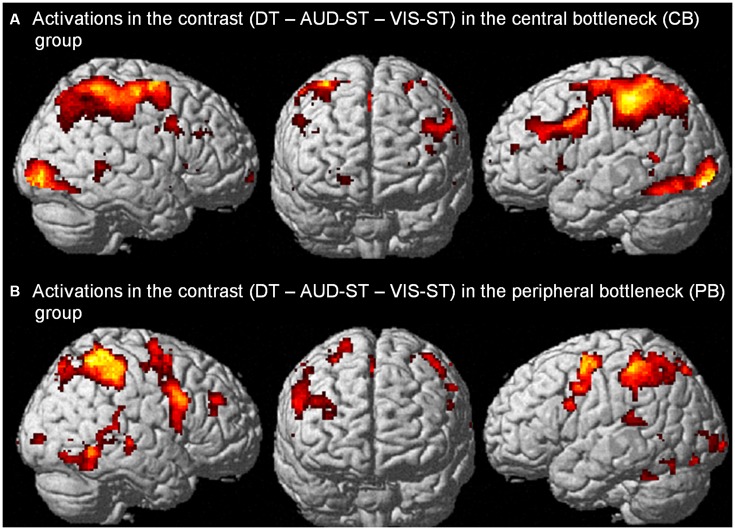
**Activations in the contrast (DT – AUD-ST – VIS-ST) in the central bottleneck (CB) group, i.e., choice response tasks (A), and peripheral bottleneck (PB) group, i.e., simple-response tasks (B).** For display purposes, a threshold of *p* < 0.00005 (uncorrected) was used in both panels.

**Figure 3:**
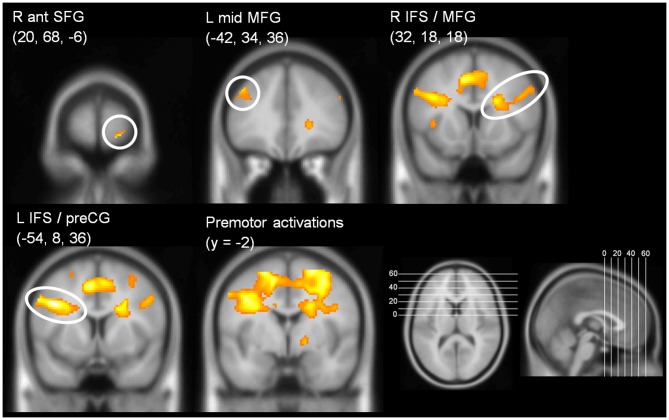
**Coronal slices illustrating prefrontal activations in the contrast (DT – AUD-ST – VIS-ST) in the CB group (i.e., choice response tasks).** *y*-coordinate of the slices is at the *y*-coordinate of the peak voxel. For display purposes, a threshold of *p* < 0.00005 (uncorrected) was used.

In the CB group, the contrast (DT – AUD-ST – VIS-ST)^7^ resulted in activations in the left and right LPFC (Figures 2A, 3, Table 1). In the left hemisphere, the middle frontal gyrus (MFG; BA 9) was activated. In addition, there was an activation spreading across the left posterior inferior frontal sulcus (IFS), posterior MFG, and the precentral sulcus (BA 9/46/6), including an area previously described as the junction point (Brass et al., 2005). In the right hemisphere, the very anterior superior frontal gyrus (SFG, BA 10/Fp1) was activated in addition to the more posterior right IFS/MFG (BA 9/46). In both hemispheres, the premotor cortices were activated extensively, including lateral precentral gyri (BA 6), posterior superior frontal gyri (SFG; BA 6), and the left posterior MFG (BA 6). Medially, the pre-supplementary motor area (preSMA; BA 6), extending posteriorly into the SMA (BA 4), and the right anterior cingulate cortex (ACC; BA 24/32) were activated. The lateral prefrontal activations tended to be stronger and more extended in the left than in right hemisphere. In addition, the anterior insula was activated bilaterally. Besides frontal areas, we also observed activation in the lateral (bilateral inferior parietal lobes (IPL), extending posteriorly along the intraparietal sulci (IPS); BAs 2/40/7) and medial (precuneus; BA 7) parietal cortices, as well as the left inferior temporal gyrus (BA 20), and posterior occipital areas in visual cortices (bilateral inferior occipital gyri extending into lingual and fusiform gyri; BAs 17/18/hOc3v). Overall, this confirms previous findings that CBs in the PRP paradigm demand executive functions in the LPFC (Marois and Ivanoff, 2005).

**Table 1:** **Areas significantly activated in the contrast (DT – AUD-ST – VIS-ST) for the central bottleneck (CB) group (i.e., choice-response tasks), thresholded at *p* < 0.05 (FWE corrected)**.

Anatomical area	BA	*x*, *y*, *z*	T	*p* (FWE)
*Frontal*
R ant SFG	10/Fp1	20, 68, −6	7.28	0.0393
L mid MFG	9	−42, 34, 36	7.14	0.0471
R IFS/MFG^C^	9/46	32, 18, 18	7.32	0.0374
L IFS/preCG^C^	9	−54, 8, 36	8.31	0.0107
L IFS/preCG	9/6	−44, 4, 34	13.39	0.0000
L post MFG	6	−54, 2, 42	7.56	0.0272
R ACC	24/32	16, 22, 28	9.11	0.0041
L preSMA	6	−8, 10, 48	10.26	0.0011
R preSMA	6	6, 10, 52	8.21	0.0121
L SMA	4	−14, −2, 54	10.18	0.0012
R SMA	4	14, 2, 52	7.92	0.0174
R preCG	6	42, −4, 30	7.23	0.0419
L preCG	6	−34, −3, 40	9.77	0.0019
L ant insula		−28, 20, 2	8.69	0.0003
R ant insula		30, 26, 4	11.10	0.0000
L SFS	6	−20, −6, 62	11.74	0.0000
R SFS	6	20, −10, 52	11.22	0.0000
*Parietal*
L IPL/IPS	2/40/7	−44, −36, 44	18.98	0.0000
R IPL/IPS	2/40/7	40, −36, 54	13.41	0.0000
L precuneus	7	−8, −72, 40	11.49	0.0000
R precuneus	7	14, −68, 46	9.94	0.0016
*Temporal*
L ITG	20	−52, −52, −18	8.39	0.0097
*Occipital*
R IOG	17/18/hOc3v	20, −92, −8	28.13	0.0000
L IOG	17/18/hOc3v	−22, −94, −6	17.95	0.0000

*Notes: Peak activations in MNI coordinates. ^C^This peak correlated significantly with behavioral DT costs. BA, Brodmann’s area; L/R, left/right hemisphere; ant/mid/post, anterior/middle/posterior; IFS, inferior frontal sulcus; MFG, middle frontal gyrus; SFG, superior frontal gyrus; ACC, anterior cingulate cortex; preCG, precentral gyrus; (pre)SMA, (pre-)supplementary motor area; IPS, intraparietal sulcus; IPL, inferior parietal lobe; ITG, inferior temporal gyrus; IOG, inferior occipital gyrus; x, y, z MNI voxel peak coordinate; p (FWE) FWE corrected p-value of the peak voxel (threshold p < 0.05)*.

**Figure 4:**
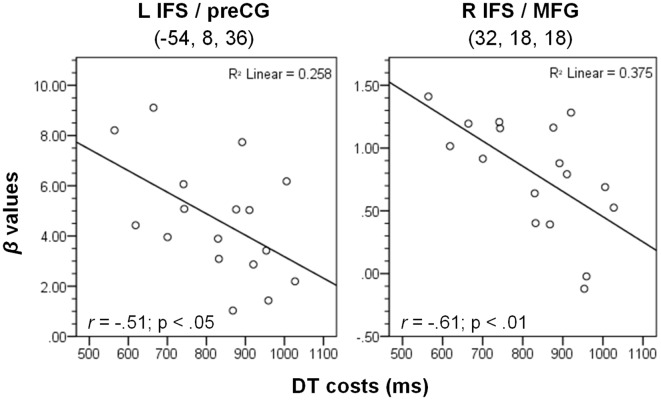
**Correlation between dual-task costs and beta-values of the contrast (DT – AUD-ST – VIS-ST) in the CB group (*N* = 17) at the two given peak coordinates (see Table 1).** L/R, left/right hemisphere; IFS, inferior frontal sulcus; MFG, middle frontal gyrus; preCG, precentral gyrus. See “Materials and Methods” and “Results” Section for more details.

To characterize the relationship between bottleneck processing and brain activity in more detail, we first calculated the behavioral dual-task costs. To ensure that all potential behavioral costs are reflected by this measure, we used the difference between dual-task RT2s and single-task RTs. In more detail, we calculated the mean RT2 of the dual-task (averaged across both orders, i.e., DT-AV and DT-VA) and subtracted the mean RT1 of the single tasks (averaged across AUD-ST and VIS-ST). These behavioral dual-task costs (829 ms) were highly significant (one-sample *t*-test vs. 0; *t*_(16)_ = 25.06, *p* < 0.001). Next, we extracted the beta-values of each participant at the location of the prefrontal activation peaks (Table 1) using the individual contrast image files as calculated in the first-level statistics for the contrast DT – AUD-ST – VIS-ST. Finally, we correlated this dual-task specific signal change with the behavioral dual-task costs (Figure 4). Pearson’s bivariate correlations revealed significant negative associations between beta-values and dual-task costs in two areas, i.e., the left IFS/MFG (peak −54, 8, 36; *r* = −0.51; *p* < 0.05; *N* = 17) and the right IFS/MFG (peak 32, 18, 18; *r* = −0.61; *p* < 0.01). Many other prefrontal peaks also showed negative correlations, but the correlations failed to reach statistical significance (*p*-values between 0.1 and 0.2). These negative correlations reflect that higher activation in the LPFC is associated with lower dual-task costs.

#### PB Group

**Figure 5:**
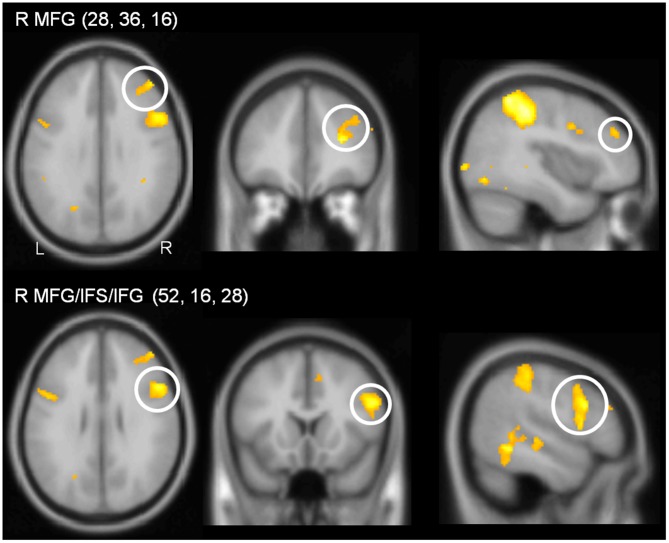
**Sections illustrating lateral prefrontal activations in the contrast (DT – AUD-ST – VIS-ST) in the PB group (i.e., simple-response tasks).** Coordinates of the sections correspond to the coordinates of the peak voxel. For display purposes, a threshold of *p* < 0.00005 (uncorrected) was used.

In the PB group, the contrast (DT – AUD-ST – VIS-ST) resulted in activations in the right LPFC (Figures 2B, 5, Table 2). In more detail, the right mid-MFG (BA 9/46) as well as the right posterior MFG (9/45) were activated. The latter activation in the right posterior MFG extended into the IFS and inferior frontal gyrus (IFG). In addition, the left precentral gyrus (BA 6) was activated. Besides frontal areas, the bilateral superior parietal cortices (BA 2/7), and the left ITG (BA 37) were activated. This demonstrates that PBs in the PRP paradigm, too, demand executive functions in the LPFC.

**Table 2:** **Areas significantly activated in the contrast (DT – AUD-ST – VIS-ST) for the peripheral bottleneck (PB) group (i.e., simple-response tasks), thresholded at *p* < 0.05 (FWE corrected)**.

Anatomical area	BA	*x*, *y*, *z*	*T*	*p* (FWE)
*Frontal*
R mid MFG	9/46	28, 36, 16	10.21	0.002
R post MFG/IFS/IFG^C^	9/45	52, 16, 28	7.84	0.031
L preCG	6	−28, −6, 54	9.25	0.005
*Parietal*
L IPS	hIP3	−36, −50, 42	9.73	0.004
L SPL	7	−8, −72, 52	8.34	0.017
L SPL	2	−32, −40, 54	8.00	0.026
R SPL/IPS	2/7/hIP3	42, −36, 48	9.57	0.005
R SPL	7	14, −62, 56	8.00	0.026
*Temporal*
R ITG	37	50, −50, −8	8.30	0.018

*Notes: Peak activations in MNI coordinates. ^C^This peak correlated significantly with behavioral DT costs. R, right hemisphere; L, left hemisphere; MFG, middle frontal gyrus; IFG/IFS, inferior frontal gyrus/sulcus; preCG, precentral gyrus; IPS, intraparietal sulcus; SPL, superior parietal lobe; ITG, inferior temporal gyrus; BA, Brodmann’s area; x, y, z MNI voxel peak coordinate; p (FWE) FWE corrected p-value of the peak voxel (threshold p < 0.05)*.

**Figure 6:**
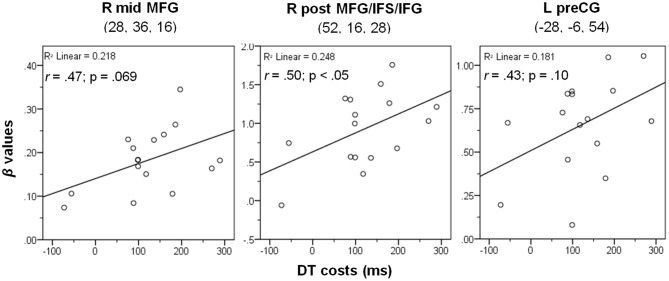
**Correlation between dual-task costs and beta-values of the contrast (DT – AUD-ST – VIS-ST) in the PB group at the three given peak coordinates (see Table 2).** Notes: L/R, left/right hemisphere; mid, middle; post, posterior; MFG, middle frontal gyrus; IFS/IFG, inferior frontal sulcus/gyrus; preCG, precentral gyrus. See Methods and Results for more details.

To characterize the relationship between bottleneck processing and brain activity in more detail, we again calculated correlational analyses as described above. Behavioral dual-task costs were calculated as DT-AV RT2 – VIS-ST (in the PB group, only the order DT-AV was used, i.e., RT2 is always the visual task). These behavioral costs in the PB task (122 ms) were reliable (one-sample *t*-test vs. 0; *t*_(15)_ = 5.06, *p* < 0.001), but significantly smaller than those in the CB group (793 ms; two-sample *t*-test; *t*_(31)_ = 12.73; *p* < 0.001). Next, we extracted the beta-values of each participant at the location of the prefrontal activation peaks (Table 2) using the individual contrast image files as calculated in the first-level statistics for the contrast DT – AUD-ST – VIS-ST. Finally, we correlated this dual-task-specific signal change with the behavioral dual-task costs (Figure 6). Pearson’s bivariate correlations revealed a significant positive association between beta-values and dual-task costs in one area: the right posterior MFG/IFS/IFG (peak 52, 16, 28; *r* = 0.50, *p* < 0.05). In addition, the positive correlations in the right mid-MFG (peak 28, 36, 16; *r* = 0.47, *p* = 0.069) and the left precentral gyrus (peak −28, −6, 54; *r* = 0.43, *p* = 0.10) were marginally significant. These positive correlations reflect that higher activation in the right LPFC is associated with higher dual-task costs.

#### Comparison of Correlations

In the CB group, prefrontal cortices correlated negatively with dual-task costs, while in the PB group the correlations were positive. This difference in the direction of the correlation between the groups was statistically significant, which necessarily follows from the fact that one group showed correlations significantly larger than zero, while the other group showed correlations significantly smaller than zero.

To rule out that this pattern is solely due to the investigated anatomical locations, which differed between the CB and PB groups, we calculated the same correlations for each group at the peaks of the respective other group. As can be seen in Table 3 (column “Correlations CB group”), correlations in the CB group where always negative, even at the coordinates derived from the peak voxels in the PB group (rows below “PB group peaks”). In the same way, correlations in the PB group (column “Correlations PB group”) were always positive, even at the coordinates derived from the peak voxels in the CB group. Although these correlations at the coordinates of the other group did not reach statistical significance, this demonstrates that the currently observed differences in the direction of the correlations are unlikely to be due to anatomical differences. This is further supported by the fact that most prefrontal CB group peaks showed negative correlations, although these failed to reach significance. Finally, note that if the dual-task costs for the CB group are calculated in the same way as for the PB group (i.e., DT-AV RT2 – VIS-ST RT1), the overall correlational pattern remains the same. Taken together, there is strong evidence that the differential correlational patterns between the CB and PB groups are caused by the experimental manipulation.

**Table 3:** **Extended analyses of the correlations between dual-task costs and beta-values in the contrasts DT – AUD-ST – VIS-ST**.

Location/Peak voxel	Correlations CB group	Correlations PB group
*CB group peaks*
R IFS/MFG (32, 18, 18)	*r* = −0.613; *p* < 0.01	*r* = 0.438; *p* = 0.088
L IFS/preCG (−54, 8, 36)	*r* = −0.508; *p* < 0.05	*r* = 0.386; *p* = 0.138
		
*PB group peaks*
R middle MFG (28, 36, 16)	*r* = −0.133; *p* = 0.612	*r* = 0.466; *p* = 0.069
R post MFG (52, 16, 28)	*r* = −0.311; *p* = 0.224	*r* = 0.498, *p* < 0.05
L preCG (−28, −6, 54)	*r* = −0.334; *p* = 0.190	*r* = 0.426, *p* = 0.100

*The column “Correlations CB group” displays the correlations between dual-task costs in the CB group and beta-values in the CB group for the peak voxel coordinates as derived from the CB group, and also at the peak voxel coordinates as derived from the PB group (“Correlations PB group” accordingly)*.

## Discussion

### Summary of Findings

To summarize, the CB group, suffering from a central bottleneck at the response selection stage, as well as the PB group, suffering from a peripheral bottleneck at the response initiation stage, both showed considerable slowing down of the second response in the dual task, i.e., they both showed dual-task costs. With respect to the imaging data, we found that the presence of a central bottleneck (CB group) resulted in activation of LPFC, mainly along the bilateral middle frontal gyri, the left posterior IFS extending into the precentral sulcus (junction point), and the right anterior SFG. In addition, lateral and medial premotor cortices were activated extensively. Higher activation in these areas was associated with lower behavioral dual-task costs (negative correlation). The presence of a PB (PB group) resulted in activation of mainly the right lateral-prefrontal cortex, again along the MFG, but posteriorly also extending into the IFG. The premotor cortex was activated bilaterally. In the PB group, higher activation in these areas was associated with higher behavioral dual-task costs (positive correlation).

### Cortical Areas Associated with Bottleneck Processing

To identify activation related to bottleneck processing, we compared the dual-task with the summed activation of the single tasks. This means that the observed activations in the LPFC are specific to the dual-task situation and cannot be explained by the summed effects of the activation in both single tasks performed separately^8^. Thus, the finding of additional dual-task specific activation indicates that additional mental processing has taken place in the dual-task compared to the single-task situation.

We propose that these additional mental demands are caused by the presence of a central (CB group) or peripheral (PB group) processing bottleneck. For both groups, we found behavioral evidence for the presence of a bottleneck in form of a considerable slowing of the second response (Pashler, 1994; Schubert, 1999). Importantly, in these situations both tasks interfere with each other, e.g., by competing for bottleneck processing. Behavioral research as well as theories of dual-task performance have suggested that this interference is resolved by executive functions (De Jong, 1995; Meyer and Kieras, 1997; Logan and Gordon, 2001; Marois and Ivanoff, 2005; Sigman and Dehaene, 2005; Schubert, 2008). In more detail, it has been suggested that the coordination of both tasks at the stage of the bottleneck might involve the inhibition of the second task until the first task has been processed by the bottleneck mechanism and the switching of the bottleneck mechanism to the second task. It is noteworthy that these detailed processes (inhibition, switching) neatly fit the general descriptions of executive functions (Miyake et al., 2000; Baddeley, 2003; Engle and Kane, 2004). Our data suggest that both, central as well as peripheral bottlenecks demand such executive functions localized in LPFC^9^.

Our findings are in agreement with a number of previous studies. In particular, the LPFC have frequently been associated with the coordination of dual-task performance (D’Esposito et al., 1995; Koechlin et al., 1999; Szameitat et al., 2002, 2006; Schubert and Szameitat, 2003; Marois and Ivanoff, 2005; Dux et al., 2006; Schubert, 2008; Stelzel et al., 2008, 2009; Tombu et al., 2011; Yildiz and Beste, 2014). Further support comes from a recent study which showed that gray matter volume in the LPFC is increased after 4 weeks of multitasking training (Takeuchi et al., 2014). In addition, our findings are supported by further neuroimaging studies which investigated the proposed processes in the context of different paradigms. For instance, we suggested that the inhibition of the second task while the first task is processed by the bottleneck is an additional demand caused by the presence of a bottleneck. Inhibition has frequently been associated with the right posterior IFS and IFG (Konishi et al., 1999; Levy and Wagner, 2011). In accordance with this, we observed activation in this area for central as well as peripheral bottlenecks.

Another demand which arises only due to the presence of a bottleneck is the requirement to constantly switch the bottleneck between the tasks. The switching between tasks and sets has been associated predominantly with left MFG and a region termed the left inferior frontal junction (IFJ), i.e., the junction of the posterior IFG/IFS with the precentral sulcus (Dove et al., 2000; Sohn et al., 2000; Brass et al., 2005). However, these areas were activated above threshold only in the CB group, while they showed only sub-threshold activation (significant at *p* < 0.0005, uncorrected) in the PB group. This suggests that the switching between the two tasks is more demanding in the CB group than in the PB group. One reason for this might be that the retrieval of two-choice-response task-sets, which is part of task switching (Monsell, 2003), is more demanding than the retrieval of simple-response task-sets.

A final demand which arises only due to the bottleneck is to ensure that the tasks are processed in the correct order. In the present study, participants always had to respond to the tasks in a given order, and they received error feedback if they had failed to do so. To ensure optimal task performance, it is necessary that also the bottleneck processes the tasks in the required order, which may involve pre-setting the bottleneck to the expected task and monitoring task performance (De Jong, 1995; Meyer and Kieras, 1997). It has been shown that this demand of task-order control is localized in the LPFC such as the left IFS and the right MFG (Szameitat et al., 2006; Schubert, 2008), areas which were also activated in the present study.

Taken together, the present findings demonstrate that central as well as peripheral bottlenecks demand additional mental processes which are localized in the LPFC and are most likely related to executive functions. This study builds upon studies which aimed at localizing brain areas that *constitute* a CB (Dux et al., 2006; Hesselmann et al., 2011; Tombu et al., 2011) and demonstrated areas that are involved as a *consequence* of the presence of a bottleneck.

### Comparison of Peripheral and Central Bottlenecks

The second aim of the present study was to provide initial evidence for potential similarities and differences in the localization of executive functions related to central and peripheral bottlenecks. As described in the “Introduction” Section, a direct comparison of the CB and PB group seems inappropriate, because the data were derived from two different fMRI scanners which may differ in their physical properties, such as signal-to-noise ratios. Consequently, differences in a direct statistical comparison of the CB and PB groups might be caused either by scanner characteristics or by the experimental manipulation. However, scanner effects are more likely to affect the whole brain relatively homogenously, rather than certain brain areas in particular. Therefore, our comparison of the two groups is restricted to a tentative qualitative comparison of the activation patterns.

One difference between the CB and PB groups which is rather unlikely to be caused by physical MRI scanner differences is the differential hemispheric weighting in the LPFC. In more detail, in the CB group, prefrontal activations were bilateral, but slightly stronger in the left hemisphere, while in the PB group, prefrontal activations were unilateral in the right hemisphere (with the exception of the precentral gyrus). However, it is noteworthy that the PB group showed subthreshold activations (at *p* < 0.005, uncorrected) also in the left lateral prefrontal cortex along the MFG (data not shown). Thus, one should exert caution in interpreting the absence of left LPFC activation in the PB group as evidence for the null hypothesis (i.e., no dual-task specific activation). For instance, increasing the sample size and statistical power might reveal activations also in the left LPFC. Future studies need to resolve this question.

On the level of gross anatomical structures, such as gyri and sulci, there is a certain overlap of the right-hemispheric activation patterns between the CB and PB groups. However, on the level of the peak voxel locations, there is usually a notable Euclidian distance of at least 20 mm between CB and PB peaks. Again, we would tentatively interpret such specific local differences as being more likely caused by differences in mental processing between the CB and PB groups, than by physical differences between the different MRI scanners used.

Taken together, the present results show that both, central as well as peripheral bottlenecks activate the LPFC. In the right hemisphere, both types of bottleneck activate comparable gross anatomical structures, but differ in their exact anatomical location. As regards the left LPFC, future studies are needed to examine whether the subthreshold activation observed in the PB group is just a non-significant random finding of the present study, or whether this activation suffers from a lack of statistical power to become significant. If the latter were the case, then central and peripheral bottlenecks would both involve the left and the right lateral prefrontal cortex, with the difference being in the relative, rather than the absolute, involvement of each hemisphere. In any case, however, the present data suggest that the brain areas associated with central and peripheral bottlenecks are non-identical, i.e., they differ at least partially. While these interpretations have to be taken with caution, due to their tentative and exploratory nature and design limitations, we contend that they nevertheless prove useful in informing future studies designed to test this issue more directly (e.g., using a within-subject design).

### Correlations Between Dual-Task Costs and Activation Strength

In both groups, we correlated behavioral dual-task costs (dual-task RT2 minus single-task RT) with a measure of neural dual-task costs (beta values of the contrast DT – AUD-ST – VIS-ST). Of note, the CB and PB groups differed in their correlational patterns: A central bottleneck (CB group) led to a negative correlation, i.e., higher activation in the LPFC was associated with lower dual-task costs, whereas a peripheral bottleneck (PB group) led to a positive correlation, i.e., higher activation in the LPFC was associated with higher dual-task costs. This suggests that the interference-resolving processes reflected in the LPFC activations are demanded differentially. One explanation of such differential demands might be derived from the findings of De Jong (1993). He argued that one major source of interference giving rise to a PB is potential crosstalk between the motor programs for the two hands (cf. also Kelso et al., 1983). We suggest that there are interindividual differences in the amount of crosstalk experienced. If crosstalk is high, dual-task costs are high and correspondingly, the demands on processes for resolving the crosstalk are high. Consequently, a positive correlation between LPFC activation (reflecting the interference resolving processes) and dual-task costs is predicted, and this is exactly what we observed.

In contrast, a central bottleneck (CB group) led to a negative correlation, i.e., higher activation in the LPFC was associated with lower dual-task costs. Such a pattern is expected to arise if higher amounts of processing are beneficial for task performance. For instance, in the related task-switching paradigm, Brass and von Cramon (2002) have shown that higher activation during a preparation period is associated with lower task-switching costs. The underlying idea is that those participants who invest a lot of processing in the task benefit in terms of better performance. In agreement with this, it has been shown for the PRP paradigm, too, that performance can benefit from preparatory processes, in particular of pre-setting the CB to the first task and preparing the switch of the bottleneck to the second task (De Jong, 1995; Luria and Meiran, 2003). Thus, we suggest that the participants with higher LPFC activation invested (consciously or unconsciously) more effort into resolving the interference, which led to lower dual-task costs.

Taken together, the results of the correlational analysis suggest that while both, central as well as peripheral bottlenecks, demand executive functions related to resolving interference at the respective bottleneck stage, either the demands on these executive functions or their exact nature are at least partially different. The nature of these differences has to be clarified in future research, but may be caused by differences in the type of interference arising from each bottleneck.

### Relation to Previous Evidence

A previous study by Herath et al. (2001) also used a PRP paradigm with simple-response tasks. However, there are some notable differences between the two studies. In particular, Herath et al. (2001) did not compare the dual-task with the summed single-task performance (contrast 1 −1 −1; Szameitat et al., 2011). Instead, they compared a sum of two dual tasks with a sum of two single tasks, which is equivalent to the comparison dual-task—mean of single-tasks (contrast 2 −1 −1). As described in detail in Szameitat et al. (2011), this contrast is not suitable for identifying dual-task-specific activation as discussed in the present manuscript. However, Herath et al. (2001) in addition used a parametric manipulation approach by introducing a long and a short SOA between the two tasks. The idea is that both tasks compete for bottleneck processing only at the short SOA, while such a competition is absent at the long SOA. When the long SOA was subtracted from the short SOA, Herath et al. (2001) also observed activation in the right lateral prefrontal cortex. In addition, this activation also correlated positively with behavioral dual-task costs. However, in more detail, the activation was localized in the ventrolateral prefrontal cortex (IFG), while we also observed strong dorsolateral prefrontal activation in the MFG. Importantly, only the latter is roughly homologous to the activations elicited by a PRP task with a CB.

The discrepancy between Herath et al. (2001) and the present study may be owing to a number of reasons. The main reason might be the contrasts employed to analyze the data. We compared the dual-task performance with single-task performance. This approach aimed at assessing all conceivable additional executive demands which may arise due to the occurrence of a bottleneck. For instance, it has been shown that already the knowledge that a dual-task will be presented on the next trial results in a number of preparatory processes, such as preparing to respond to the tasks in the expected order by pre-setting the bottleneck mechanism (De Jong, 1995; Luria and Meiran, 2003). Such processes, which have been localized, among other areas, in the right MFG (Szameitat et al., 2006), might not have been captured by Herath et al. (2001) because they compared two DT with each other which might both involve such preparatory processes.

It is interesting to note that one main argument made by Herath et al. (2001) is based on the use of two fingers of the same hand for responding to both stimuli. This resulted in an overlap of motor cortex activation of the two tasks. Based on the cortical-field hypothesis (Roland and Zilles, 1998), Herath et al. (2001) argued that this overlap results in interference between both tasks (cf. also Nijboer et al., 2014; Alavash et al., 2015; Salo et al., 2015). The present study challenges this interpretation because we observed similar interference and cortical activations even though participants used one finger of each hand to respond to the two simple-response tasks, i.e., motor cortex activation did not overlap. Instead, we suggest that the interference arises due to a PB at the stage of the response initiation. Whether such a bottleneck is also localized in LPFC, as has been shown for CBs (Dux et al., 2006; Tombu et al., 2011), remains to be established.

Taken together, in a more global view, Herath et al.’s (2001) and the present findings are in agreement with each other, because they both showed PB-related activation in the right lateral prefrontal cortex, and the activation in these right-hemispheric areas correlated positively with behavioral dual-task costs. The differences between the studies are most likely due to design and analysis differences, as our approach potentially captured more executive functions involved in task processing suffering from PBs.

## Conclusion

Taken together, we were able to show that peripheral as well as central processing bottlenecks activate the LPFC. We suggest that these dual-task specific activations are most likely associated with executive functions coordinating task processing at a central (CB group) or peripheral (PB group) bottleneck. The differential hemispheric weighting and the differences in the peak coordinates of these activations in combination with the differential correlational patterns between activation strength and dual-task costs suggest that the involved executive functions are demanded differently. One reason why these findings are highly relevant is the pervasiveness of these bottlenecks: even the concurrent performance of probably the most simplistic tasks results not only in performance decrements, but also demands executive functions. Importantly, executive functions are considered to be a limited resource themselves (Barrouillet et al., 2004; Engle and Kane, 2004). Thus, if executive functions are occupied by coordinating task processing at a bottleneck, they are not available for other tasks. This is relevant for multitasking activities where lapses in higher-level action control may have serious consequences, such as driving a car or in certain occupations such as air traffic controllers.

## Author Contributions

The study was designed by AJS, AV, and HJM. The data were acquired and analyzed by AJS and AV. The data were interpreted by AJS, AV, and HJM. The manuscript was written by AJS, AV, and HJM.

## Conflict of Interest Statement

The authors declare that the research was conducted in the absence of any commercial or financial relationships that could be construed as a potential conflict of interest.

## Footnotes

^1^In the present context terms like *inhibition* or *switching* refer to cognitive processes. For instance, whether cognitive inhibition is physiologically implemented by inhibitory synaptic transmission is unclear and not in the scope of the present manuscript.

^2^Please note that this research question does not aim at understanding the bottleneck itself (e.g., its cause or functional neuroanatomical correlates), but instead at understanding the mental processes arising as a consequence of the presence of a bottleneck.

^3^http://www.brunel.ac.uk/courses/postgraduate/functional-neuroimaging-msc

^4^http://www.psy.lmu.de/ncp/

^5^http://www.pc.rhul.ac.uk/sites/CUBIC/

^6^http://www.fil.ion.ucl.ac.uk/spm/software/spm12/

^7^In detail, there were two DT conditions which differed in the presentation order of the tasks [DT-AV (auditory task first) and DT-VA (visual task first)]. These two conditions were modeled separately and averaged for analysis, resulting in the contrast [(DT-AV + DT-VA)/2 – AUD-ST – VIS-ST], i.e., [0.5 0.5 −1 −1].

^8^Note that we claim that the observed activation is specific for the dual task. We do not claim that the cortical areas are specific for dual-task processing. They may be involved in other processing as well, incl. processing of the single tasks.

^9^Note that the claim that the dual-task specific activations in the LPFC are related to executive functions is an indirect one, based on combining our current observations with previous knowledge about the functionality of the activated areas on the one hand and the cognitive processes known to be at work during bottleneck processing on the other hand. The current study cannot directly prove that the LPFC activations are caused by executive functions.
